# Synthetic bionanotechnology: synthetic biology finds a toehold in nanotechnology

**DOI:** 10.1042/ETLS20190100

**Published:** 2019-10-23

**Authors:** Alexander A. Green

**Affiliations:** Biodesign Center for Molecular Design and Biomimetics at the Biodesign Institute and School of Molecular Sciences, Arizona State University, Tempe, Arizona 85287, U.S.A.

**Keywords:** nucleic acid nanotechnology, RNA, synthetic biological circuits

## Abstract

Enabled by its central role in the molecular networks that govern cell function, RNA has been widely used for constructing components used in biological circuits for synthetic biology. Nucleic acid nanotechnology, which exploits predictable nucleic acid interactions to implement programmable molecular systems, has seen remarkable advances in *in vitro* nanoscale self-assembly and molecular computation, enabling the production of complex nanostructures and DNA-based neural networks. Living cells genetically engineered to execute nucleic acid nanotechnology programs thus have outstanding potential to significantly extend the current limits of synthetic biology. This perspective discusses the recent developments and future challenges in the field of synthetic bionanotechnology. Thus far, researchers in this emerging area have implemented dozens of programmable RNA nanodevices that provide precise control over gene expression at the transcriptional and translational levels and through CRISPR/Cas effectors. Moreover, they have employed synthetic self-assembling RNA networks in engineered bacteria to carry out computations featuring up to a dozen inputs and to substantially enhance the rate of chemical synthesis. Continued advancement of the field will benefit from improved *in vivo* strategies for streamlining nucleic acid network synthesis and new approaches for enhancing network function. As the field matures and the complexity gap between *in vitro* and *in vivo* systems narrows, synthetic bionanotechnology promises to have diverse potential applications ranging from intracellular circuits that detect and treat disease to synthetic enzymatic pathways that efficiently produce novel drug molecules.

RNA plays a pivotal role in the molecular networks that govern cellular function [1,2], serving as the template for translation and taking on multiple regulatory and sensing functions to modulate gene expression. For these reasons, RNA has been widely used for constructing components used in biological circuits for synthetic biology. RNA devices can provide regulation at the level of transcription [3,4] or translation [5–7]. Moreover, they can be engineered in the form of riboswitches [8] to regulate gene expression in response to different small molecules and proteins. Over more than a decade, these advances in RNA synthetic biology have led to a plethora of RNA-enabled circuits that endow cells with unnatural functions, enabling them to perform logic [3,4,9,10], detect combinations of small molecules [11], and even identify cancer cells [12].

In parallel with these developments, the field of nucleic acid nanotechnology, which exploits the predictable base-pairing properties of DNA and RNA for implementing molecular systems, has seen remarkable advances in nanoscale self-assembly and molecular computation. Using DNA-based self-assembly methods, it is now possible to construct three-dimensional structures of virtually any geometry with nanometer-scale resolution using hundreds or even thousands of programmed DNA strands [13–15]. The use of strand-displacement systems, where single-stranded regions known as toeholds are used to promote hybridization reactions that release new interaction domains, has enabled test tube implementations of square root calculators [16] and neural networks [17] capable of pattern recognition using hundreds of strands [18]. Chemically synthesized DNA and RNA have been transfected into fixed zebrafish embryos for multiplexed endogenous RNA imaging [19] and live cells for logic operations and siRNA activation [20] using strand-displacement reactions. In addition, the Pierce laboratory has focused on the development of conditional RNAi systems that have potential as programmable drugs activated in response to RNA inputs [21,22]. While conditional RNAi systems have not been demonstrated in living cells, they have provided a valuable testbed for studying the effects of RNA degradation, target site accessibility, and different interaction motifs that could play important roles for *in vivo* RNA networks.

The fact that these impressive feats of molecular design, assembly, and computation have made use of biomolecules suggests that cells themselves can be genetically engineered to take advantage of nucleic acid nanotechnology and significantly expand the existing capabilities of synthetic biology. For instance, nucleic acid nanostructures assembled *in vivo* could be used to construct synthetic organelles that localize enzymes and constrain reactant flux for highly efficient synthesis of valuable chemicals. Moreover, genetically encoded neural networks could lead to single cells with the computing power of multiple neurons and the ability to respond intelligently to changes in environmental conditions. Such information-processing systems also have the long-term potential for upload into human cells to passively scan the transcriptome and intervene only if cellular dysfunction is detected by producing a drug or silencing a pathway.

Realizing these synthetic bionanotechnology systems, however, requires addressing multiple technical challenges. First, the bulk of work in nucleic acid nanotechnology has focused on DNA molecules with exposed single-stranded domains to initiate reactions [23,24]. DNA in cells typically exists in double-stranded form and single-stranded DNA is relatively hard to synthesize *in vivo*. Thus, systems implemented in live cells have mostly relied on RNA molecular networks, which have distinct properties from their *in vitro* DNA counterparts. Second, nucleic acid nanotechnology systems often employ strands with well-defined 5′ and 3′ ends and partially double-stranded complexes. *In vivo* systems implemented with transcribed RNA, however, often require specific sequence elements at their ends, such as terminator hairpins and 5′ untranslated regions. Partially double-stranded complexes between two RNAs are also not guaranteed to assemble in the cell. Third, most self-assembled nucleic acid nanostructures are formed using annealing procedures [13–15], where the strands are heated to ∼80°C and slowly cooled so that they reach their thermodynamically optimum assembled state. Most cells would not survive these temperature treatments and thus *in vivo* systems are limited to isothermal nucleic acid reactions. They may also need to integrate cotranscriptional folding pathways, which have been harnessed for *in vitro* assembly of RNA origami nanostructures [25]. Fourth, *in vivo* nucleic acid circuitry must be coupled to an observable cellular output that is genetically encoded, such as a reporter protein, enzyme, or aptamer, to assess circuit function and alter cell behavior. Most *in vitro* circuits rely on DNAs with chemically synthesized fluorophore/quencher pairs [23] and in other cases DNA aptamers or DNAzymes [26] for output. Accordingly, substantial work in synthetic bionanotechnology has focused on developing systems that detect cognate RNA sequences or particular RNA configurations and report their status via fluorescent reporter proteins, enzymes, and aptamers.

One of the earliest major works using genetically engineered cells for RNA nanostructure self-assembly was reported by the Silver laboratory and employed RNA as a programmable scaffold for proteins [27]. In this work, Delebecque et al. used a pair of interacting RNA molecules that assembled isothermally in the cytoplasm to form periodic RNA nanostructures presenting numerous peptide-binding aptamer sites. Split green fluorescent protein (GFP) reporters and enzymes fused to RNA-binding peptides were used to assess the assembly of the RNA nanostructure *in vivo*. They found that the expression of optimized RNA scaffolds was associated with an increase in the rate of hydrogen production in a two-enzyme pathway by 48-fold in engineered *E. coli* cells. This work has since been extended for scaffolding a four-enzyme pathway [28] and the assembly of genetically encoded DNA nanostructures has also been demonstrated using reverse transcriptases in *E. coli* [29].

Beyond structural applications, our laboratory and others have demonstrated the use of synthetic bionanotechnology for sensing and logic in living cells. Much of this work has been enabled by the sequence design package NUPACK developed for nucleic acid nanotechnology systems [30,31]. NUPACK not only designs nucleic acid strands that fold into a user-specified secondary structure, but it can also carry out multi-objective design where multiple strands and their complexes are optimized simultaneously. The latter capability is particularly important for designing genetically encoded RNA systems where biologically conserved elements, such as genes, the Shine-Dalgarno sequence, and terminators, must be present on interacting transcripts.

Using NUPACK, we developed a fully *de*-*novo*-designed RNA-based prokaryotic translational regulator called the toehold switch [32] inspired by the toehold-mediated strand-displacement motifs pioneered in nucleic acid nanotechnology (Figure 1A). In toehold switches, regulation is carried out by a switch RNA that features a single-stranded toehold domain followed by a hairpin structure and the coding sequence of an output gene (Figure 1B). The hairpin structure is engineered to conceal the RNA sequence elements required for translation initiation, the ribosome binding site (RBS) and the start codon, and thus represses translation of the output gene. When a cognate trigger RNA is expressed by the cell, it initiates binding through the exposed toehold domain and proceeds to unwind the hairpin stem in a strand-displacement reaction. The newly exposed RBS and start codon are then free for ribosome binding, enabling the active translation of the output gene. The use of a toehold-mediated interaction, as opposed to loop-mediated interactions used in natural systems, enabled the toehold switches to operate in *E. coli* with stronger thermodynamics and improved kinetics, helping them achieve changes in gene expression above 600-fold, a level similar to protein-based regulators and over an order of magnitude improvement compared with previous riboregulators. The design of the toehold switch allowed the sensors to detect virtually any trigger sequence, which we exploited to detect endogenous transcripts and construct libraries of dozens of sensors with low cross-talk. Using toehold-mediated designs, the Lucks group has also demonstrated that nucleic acid nanotechnology principles can be applied to transcriptional regulation. Computer-designed Small Transcription Activating RNAs (STARs) employ toehold-mediated interactions to prevent formation of a transcriptional terminator, enabling programmable control of downstream transcription (Figure 1C). These devices provided impressive ∼9000-fold activation of gene expression and lower signal leakage than toehold switches. Translational repressors that turn off gene expression upon trigger RNA binding via toehold-mediated strand-displacement reactions have also recently been reported [34,35].

**Figure 1.**
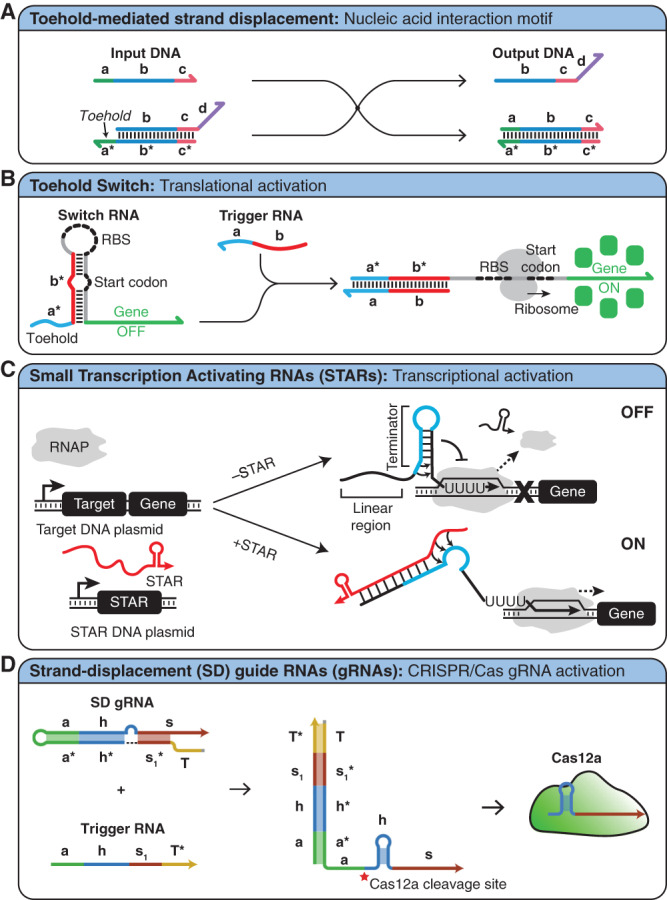
Toehold-mediated strand-displacement systems implemented in *E. coli*. (**A**) Schematic showing a toehold-mediated nucleic acid interaction. The two-strand complex has an exposed toehold domain a* that is complementary to the domain **a** in the input DNA. The input DNA binds to the complex and strand displacement through domains **b** and b* leads to release of an output DNA that can be used for downstream DNA reactions. (**B**) The toehold switch translational riboregulator [32] employs a 5′ toehold domain in the switch RNA to initiate binding with a cognate trigger RNA. Following binding between the complementary **a** and a* domains, the switch RNA stem is disrupted revealing the RBS and start codon. The newly exposed RBS and start codon can then be recognized by the ribosome for translation of the output gene. (**C**) Small Transcription Activating RNAs (STARs) use toehold-mediated interactions to bind upstream of a transcriptional terminator and prevent formation of its stem–loop secondary structure to enable transcription of the downstream gene. Adapted from Chappell et al. [33] (**D**) A strand displacement (SD) guide RNA (gRNA) employs a hairpin structure to prevent Cas12a access to the gRNA handle (**h**) and spacer (**s**) sequences. A 5′ toehold domain **T** enables binding by the trigger RNA and the ensuing strand-displacement reaction exposes both the handle and spacer to enable recognition by Cas12a. Adapted from Oesinghaus and Simmel [36].

The ability of CRISPR/Cas enzymes to regulate gene expression with a wide dynamic range in hosts ranging from bacteria to humans has made them an important focus area for synthetic bionanotechnology. Recently NUPACK has been used for generating several conditional guide RNA implementations for the enzymes Cas9 and Cas12a that respond to binding of a cognate trigger RNA. Siu and Chen demonstrated toehold-mediated activatable guide RNAs for Cas9 that operated in *E. coli* [37], while Oesinghaus and Simmel implemented activatable guide RNAs for Cas12a in the same organism [36] (Figure 1D). The Pierce laboratory developed conditional Cas9 guide RNAs whose function can be turned on or off in response to trigger RNA binding [38]. These systems operated successfully in *E. coli* and mammalian cells.

Having an established set of orthogonal, genetically encoded systems to detect transcripts in live cells has opened up new possibilities for cellular computing systems. Recently, we have reported RNA-based logic circuits [39] that can take advantage of predictable RNA–RNA interactions to evaluate Boolean logic in *E. coli* and are considerably more genetically compact than equivalent protein-based circuits. These ribocomputing circuits employ toehold switches, strand-displacement, and RNA self-assembly to encode AND, OR, and NOT logic functions that operate successfully in the cytoplasm (Figure 2A). NOT logic expressions are implemented via antisense RNAs that bind to and deactivate trigger RNAs. AND logic expressions employ assemblies of input RNAs to form a functional RNA trigger that can be detected by toehold switches. The central processing element of the ribocomputing devices is an extended transcript called a gate RNA that features multiple toehold switch hairpin modules arrayed upstream and in-frame of a common reporter protein. The gate transcript can activate translation in response to cognate RNAs from each toehold switch hairpin and thus implements OR logic. Using ribocomputing devices, we successfully implemented several multi-input logic operations and ultimately evaluated the complex disjunctive normal form expression (A1 AND A2 AND NOT A1*) OR (B1 AND B2 AND NOT B2*) OR (C1 AND C2) OR (D1 AND D2) OR (E1 AND E2). This 12-input expression represents one of the most complex synthetic logic expressions realized in a living cell. RNA-based AND logic devices have also been implemented using STARs with the formation of a two-strand small RNA complex harnessed to activate transcription [33] (Figure 2B). A similar strategy has been applied using a conditional Cas12a guide RNA and a pair of cognate trigger RNAs to implement Cas12a-mediated transcriptional repression [36] (Figure 2C). In addition, translational repressor systems have been incorporated into ribocomputing devices for multi-input logic. These repressor-based RNA circuits enable efficient implementations of the universal NAND and NOR logic operations as well as the expression NOT((A1 AND A2) OR (B1 AND B2)) in *E. coli* [34].

**Figure 2.**
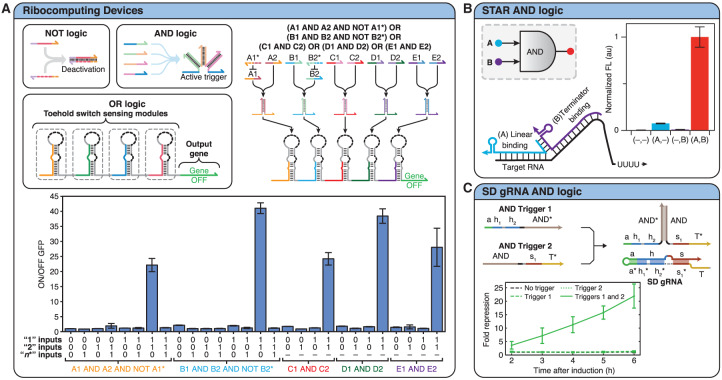
RNA-based logic circuits that regulate gene expression in *E. coli*. (**A**) Ribocomputing devices make use of RNA self-assembly and strand-displacement reactions for *in vivo* logic. Antisense interactions and formation of multi-arm junctions are used for NOT and AND operations, respectively. OR logic is performed by a gate RNA with multiple toehold switch modules integrated upstream of the output gene. These basic interaction motifs can be integrated into complex logic expressions, such as a 12-input disjunctive normal form expression that was successfully executed in live cells. Adapted from Green et al. [39] (**B**) STARs can be used to implement two-input AND logic using a pair of small RNAs that hybridize to one another to prevent the formation of a transcriptional terminator secondary structure. Adapted from Chappell et al. [33] (**C**) Conditional SD gRNAs can be activated using a pair of cognate trigger RNAs that hybridize to one another. Formation of the two-strand trigger complex generates a sequence that can interact with the SD gRNA via strand displacement to enable recognition by dCas12a, a version of Cas12a with abrogated DNase activity. The resulting gRNA/dCas12a complex can then be used to repress reporter gene expression. Adapted from Oesinghaus and Simmel [36].

Outside of the cell, toehold switches have also been incorporated into portable diagnostic devices for detection of infectious disease. These tests make use of cell-free transcription–translation systems embedded into paper substrates to detect amplified transcripts using genetically encoded pathogen-specific toehold switches [40]. The resulting paper-based diagnostics employ convenient isothermal reactions, provide test results that can be read by eye using an enzymatic reaction, and cost ∼$3 per sample [41,42].

Despite recent progress in synthetic bionanotechnology, there remains a substantial gap between the complexity of nucleic acid systems implemented *in vitro* compared with those realized in living cells. For DNA nanostructures, tile approaches have been used to assemble nanostructures consisting of 66 unique tiles at physiological 37°C temperatures *in vitro* [43]. In contrast, genetically encoded DNA nanostructures with at most four unique tiles have been assembled in *E. coli* [29]. For molecular computing, a neural network capable of recognizing nine handwritten digits was implemented using strand-displacement circuitry *in vitro* [18]. This system was implemented using a set of 205 processing and output species that interact with 100 potential input DNAs specifying the character shape. The most complex ribocomputing device deployed in *E. coli* consisted of 12 potential input RNAs and a single gate RNA, which combined five toehold switch modules and *GFP* for processing and output [39] (Figure 2A).

Fortunately, the gap between *in vitro* and *in vivo* systems provides a clear roadmap marking the capabilities required to extend the limits of synthetic bionanotechnology. Some critical future advances and research directions include:

Efficient RNA circuit synthesis: More complex circuits will require larger numbers of unique RNA species to be transcribed *in vivo*. Given the short length of DNA/RNA species employed in the most complex *in vitro* circuits, the use of promoter/terminator pairs to encode each species is an inefficient means of genetic encoding. Accordingly, future circuits will benefit from the use of efficient self-cleaving ribozymes [44] or programmed RNA cleavage sites [45] to divide long transcripts into the required circuit components (Figure 3A). On the other hand, *in vivo* RNA systems could employ alternative strategies where multiple processing elements are incorporated into a single transcript, analogous to the gate RNAs in ribocomputing devices [34,39]. Along these lines, single-stranded DNA and RNA folding schemes [25,46] can be used to assemble intracellular nanostructures. Successful implementation of systems featuring such long transcripts will benefit by integrating our growing understanding of cotranscriptional folding processes into the design process [47].

**Figure 3.**
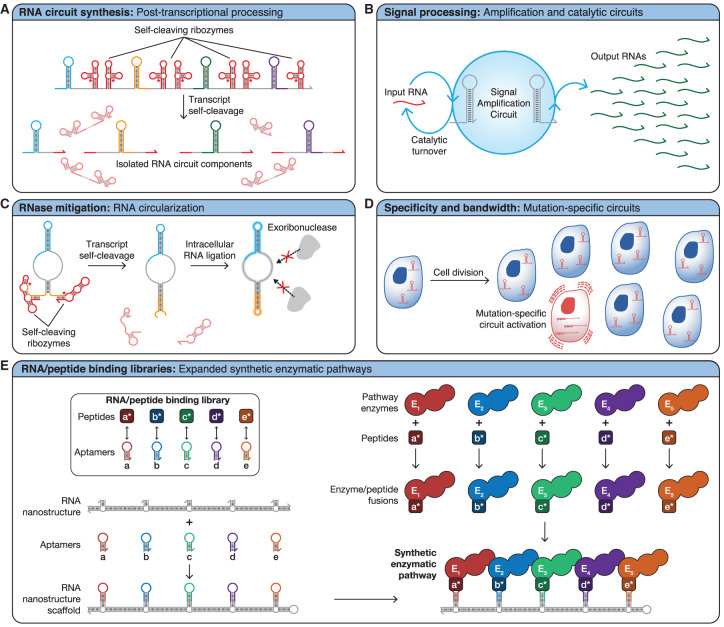
Critical future capabilities and research directions for synthetic bionanotechnology. (**A**) More complex RNA-based logic circuitry demands improved methods to synthesize RNA circuit components. Self-cleaving ribozymes can be used to generate isolated RNA components post-transcriptionally without assistance from proteins. (**B**) Enhanced signal processing capabilities *in vivo* will be required to engineer cells with more sophisticated synthetic functions. Circuits that treat endogenous transcripts as catalysts and provide amplification are essential for systems that will interface with the native transcriptome. (**C**) RNase mitigation strategies are valuable for implementing RNA-based circuits that require sequential strand-displacement reactions. RNA circularization using pairs of self-cleaving ribozymes and endogenous ligases can prolong RNA lifetime by imparting exoribonuclease resistance. (**D**) RNA-based devices with enhanced sequence specificity can increase circuit bandwidth and enable genetically encoded monitoring systems that are only activated when a specific mutation is identified. (**E**) Expanded libraries of RNA aptamers and peptides that bind to one another will be important for positioning proteins on intracellular RNA scaffolds for new enzymatic pathways and synthetic organelles. Such RNA/peptide pairs should be small in size, orthogonal, and bind with high affinity.

Enhanced signal processing: DNA components that implement signal amplification, rectification, thresholding, and annihilation are important elements in neural network circuits [17,18]. Parallel systems that function effectively *in vivo* will need to be developed to implement circuits that operate with comparable complexity in living cells. RNA signal amplification systems in which an input RNA is used catalytically are also valuable for detection of low-copy transcripts (Figure 3B) and have recently been applied to live-cell RNA imaging in *E. coli* [48]. Such catalytic systems should also minimize the likelihood that synthetic RNA networks will interfere with the endogenous transcriptome.

RNase mitigation strategies: Genetically encoded *in vivo* RNA circuits have generally been limited to a single strand-displacement step [39], whereas sophisticated *in vitro* networks can require five or more sequential strand-displacement steps [18], which requires increased reaction time. RNA systems in living cells will likely need to incorporate techniques like tRNA scaffolds [49], self- cleaving ribozyme sites [44], circularized transcripts [50], or computationally designed tertiary structures [51] to reduce degradation by RNases and prolong their lifetimes (Figure 3C). Since protective elements can require base pairing between the 5′ and 3′ ends of the transcript, new RNA–RNA interaction schemes may be necessary if the protection methods preclude or impede toehold-mediated interactions.

Enhanced specificity and bandwidth: In principle, the use of RNA for *in vivo* signal processing could enable networks with hundreds or even thousands of unique RNAs to function simultaneously in the cell. Implementing systems with maximum bandwidth will require RNA circuitry with specificity down to the single-nucleotide level. *In vitro* toehold exchange probes and related systems have demonstrated impressive specificity [52–54]. Intracellular systems with these capabilities could also be used to monitor the emergence of mutations as cells evolve or as cancer develops (Figure 3D).

Expanded RNA/peptide binding libraries and enclosed nanostructures: Genetically encoded RNA nanostructures that assemble *in vivo* can act as programmable scaffolds directing the assembly of proteins and enzymes within the cell to establish new enzymatic pathways (Figure 3E). As the complexity of these nanostructures increases, connecting elements between RNA and protein are required to ensure that such ribonucleoprotein complexes assemble as needed into organelle-like structures. Expanded libraries of RNA aptamers that bind to short peptides are essential for these purposes. It is critical that these RNA/peptide pairs exhibit orthogonal binding properties, high affinity, and are compact to minimize their potential impact on RNA assembly and protein function. Recent studies have also demonstrated [55,56] that enzyme proximation is insufficient to explain the activity enhancements observed for enzymes scaffolded by nucleic acid nanostructures and that factors such as local pH and reduced substrate diffusion can yield long-term improvements in pathway activity. Thus, future efforts should also focus on reducing substrate diffusion by implementing enclosed structures, possibly with the assistance of membrane lipids and proteins, and by constructing ‘virtual compartments’ that establish attractive interactions between substrate molecules and the nanostructured scaffold [55].

In conclusion, nucleic acid nanotechnology is providing synthetic biologists with a powerful set of molecular tools that can be harnessed to provide living cells with new functions. Genetically encoded nucleic acids have thus far been used *in vivo* to construct nanostructures that lead to enhanced enzymatic activity, sensors that detect diverse transcripts, and logic devices that process up to a dozen different input molecules. In the coming years, it is likely that future major developments will arise from RNA circuitry powered by sequential strand-displacement reactions, which will enable critical signal amplification and rectification capabilities, and related systems that will interface directly with endogenous transcripts to monitor and manipulate cell state. Such systems can be deployed by bacteria in the environment for monitoring, reporting, and remediation efforts, or even those housed in the gut where they can respond to gastrointestinal ailments or changes in nutrition. Increasingly sophisticated *in vivo* nucleic acid assembly methods will be harnessed to position larger numbers of enzymes and to fabricate synthetic organelles that replicate the compartmentalization afforded by natural systems. In the absence of effective compartmentalization schemes, however, efforts should be directed toward pathways in which reactants cannot be lost to diffusion. Possible targets include signaling proteins [57] along with nonribosomal peptide synthetases and polyketide synthases [58], which could enable biosynthesis of entirely new molecules with therapeutic potential. Lastly, genetically encoded RNA systems will increasingly move into mammalian cells, where they can ultimately be deployed to improve human health. Synthetic bionanotechnology thus has a host of promising potential applications in areas such as industrial chemical production, environmental monitoring, and medicine that rely on the ability of living cells to replicate and execute carefully engineered nucleic acid nanotechnology programs. The success of these future efforts will hinge on our ability to devise sophisticated nucleic acid systems that can be genetically encoded and are sufficiently robust to operate in the complex and dynamic intracellular environment.

## Summary

- Nucleic acid nanotechnology is providing synthetic biologists with a powerful set of molecular tools that can be harnessed to provide living cells with new functions.
- Genetically encoded nucleic acids have been used *in vivo* to construct nanostructures to scaffold enzymes, sensors that detect diverse transcripts, and logic devices that process up to a dozen different input molecules.
- It is likely future major developments will arise from RNA networks powered by sequential strand-displacement reactions, increasingly sophisticated *in vivo* nucleic acid assembly methods, and further development of circuitry for mammalian cells.
- These future efforts will require multiple new capabilities including improved methods for expressing nucleic acid networks and novel techniques for enhancing network stability, sensitivity, specificity, and function.

## Abbreviations

GFP: green fluorescent protein
RBS: ribosome binding site
SD: strand displacement
STARs: Small Transcription Activating RNAs
